# Norovirus: A Masked Relationship With Inflammatory Bowel Disease

**DOI:** 10.7759/cureus.93359

**Published:** 2025-09-27

**Authors:** Saba Sattar, Rehan Ali, Hassan Murtaza, Muhammad Qasim Qureshi, Shayan Butt

**Affiliations:** 1 Internal Medicine, King Edward Medical University, Lahore, PAK; 2 Internal Medicine, Sahiwal Medical College, Sahiwal, PAK; 3 Internal Medicine, Mayo Clinic, Jacksonville, USA

**Keywords:** bloody diarrhea, crohn's disease (cd), ibd flare, norovirus, ulcerative colitis (uc)

## Abstract

In this narrative review, we discuss the association between norovirus infection and one of the most debilitating gastrointestinal pathologies: inflammatory bowel disease (IBD), comprising ulcerative colitis and Crohn's disease. Norovirus is the leading cause of viral gastroenteritis worldwide, and IBD is an autoimmune disorder resulting from an interplay of environmental factors, genetics, and the gut microbiome. We reviewed updated information from PubMed and Google Scholar to discuss various mechanisms of this association, from Norovirus being a trigger and second hit in the genetically predisposed patients to causing a flare-up of IBD. Norovirus causes immune dysregulation as well as changes in intestinal Paneth cells. By exploring this literature, we emphasize the importance of preventing Norovirus through the development of vaccines, as well as the rapid diagnosis, management, and eradication of infections. A crucial purpose of this review article is not only to gain a better understanding of the pathology but also to highlight the need for further prospective studies to establish a causal relationship, which can help establish novel treatment protocols to enhance patient outcomes.

## Introduction and background

Norovirus, a member of the family of positive-sense, nonenveloped RNA viruses known as Caliciviridae, is among the leading enteric viruses causing sporadic cases as well as epidemic outbreaks of gastroenteritis. Approximately 20% of all acute gastroenteritis cases worldwide are attributed to norovirus. In the United States alone, human noroviruses are responsible for approximately 20 million cases of acute gastroenteritis each year. The combination of norovirus causing infection with as few as <20 particles and prompting a high yield of viral shedding from infected individuals is the primary cause of morbidity and mortality associated with its epidemics, more so in developing parts of Asia and Africa. Norovirus causes a self-limiting disease that spreads through the fecal-oral route and aerosols of vomitus. The disease course subsides in two to three days, except in immunosuppressed individuals, children, and the elderly, where it can last longer [1].

Norovirus has been studied for its association with inflammatory bowel disease (IBD), as well as other enteric infections. IBD, comprising Crohn's disease (CD; whole intestinal tract) and ulcerative colitis (UC; colon and rectum), is one of the most debilitating autoimmune gastrointestinal pathologies, causing diarrhea, abdominal pain, nausea, and fatigue. Etiology, still under research, is a combination of environmental triggers, autoimmunity, and intestinal dysbiosis. Its association with enteric infections is two-way, with the immunosuppressants used for its treatment paving the way for opportunistic infection. In contrast, enteric infections cause gut dysbiosis, thereby increasing the incidence of IBD [2].

Recent years have seen a significant increase in literature demonstrating this correlation, with a positive association between the occurrence of IBD and a positive stool test for various pathogens, including bacteria and viruses such as norovirus [3]. The mechanism of this association, primarily studied in murine samples due to obvious ethical reasons, is based on viruses causing alterations in intestinal Paneth cells, as well as stimulating interferon and interleukin pathways, ultimately leading to immune dysregulation and inflammation in IBD. Moreover, noroviruses are also studied to disrupt gut microbiota composition, thus making it favorable for autoimmune diseases like IBD [4].

In this article, we will review the literature available on the association between enteric viruses, such as Norovirus, and the development of IBD, as well as the mechanisms understood to date that cause this association.

## Review

We searched the available literature on PubMed and Google Scholar using the keywords "inflammatory bowel disease", "Crohn disease", "colitis, ulcerative", "norovirus", "caliciviridae infections", "norovirus infection", "norovirus complication". Our search yielded a total of 71 studies. We excluded non-English language publications and the articles that were not discussing anything related to the pathophysiology, etiology, or association between IBD and norovirus.

According to the review of published human studies, the incidence of an association between Norovirus and IBD is variable (Table 1). The original epidemiological study by Gebhard et al. evaluated norovirus and rotavirus (RV) antigens in stool samples from IBD patients, along with serological analysis, over 14 months [5]. Among 77 patients, 65 relapses were seen in 54 patients. Although no significant correlation existed between enteric viruses and IBD relapse, five out of eight patients with serological evidence of infection relapsed within three months (p < 0.01) [5]. In another case-control study, Khan et al. retrospectively analyzed 2,666 pediatric stool specimens that were positive for Norovirus over 10 months [6]. They found nine cases of IBD relapse (one CD, eight UC) with norovirus presence, demonstrating a significant association with UC flare-ups (p < 0.01). Some of them were also coinfected with other viruses or *Clostridium difficile*. They also concluded that Norovirus causes hematochezia when associated with IBD, compared to when it occurs alone [6].

In contrast, another study that screened 50 fecal specimens during endoscopy did not detect enteric viruses during IBD flares but did identify traces in non-IBD cases [7]. Similarly, a cohort study involving 286 IBD patients showed a low prevalence of enteric viruses and no apparent association with disease activity [8]. The most extensive study, involving 13,231 stool samples from 9,403 individuals, compared 577 patients with IBD (277 with CD and 300 with UC) with 8,826 non-IBD patients. The results showed that enteric viruses, such as Norovirus, as well as Enteroinvasive *Escherichia coli*, were more common in patients with IBD, specifically, during flare-ups of CD and UC. This was statistically significant (p < 0.001), indicating a strong correlation between these infections and the aggravation of IBD symptoms [4,9]. Similarly, Ostrowski and Croft retrospectively analyzed 171 episodes of acute severe UC, 147 of which had fecal polymerase chain reaction testing done. Enteric viral infection was diagnosed in 33 patients 37 times (24 noncytomegalovirus, predominantly adenovirus) [10]. More recently, Hassan et al. conducted a cross-sectional observational study in which 95 patients underwent stool testing, and 78 had active IC flare-ups. Assessment of the severity and extent of inflammation was done based on the Mayo score and Montreal classification. The diagnosis of UC was based on clinical, colonoscopic, and histopathological findings. Among these, enteric infections were identified in 75 patients. However, only 7.7% were caused by the enteric viruses, and Norovirus was only found in 3.8%, implying a poor correlation with UC exacerbations [11].

These discrepant findings suggest that in humans, Norovirus is often concordant with IBD flares (perhaps due to overlapping symptoms), and it may also act as a precipitating "second hit" in genetically susceptible hosts. Notably, case‐control and database studies have also determined Norovirus‐associated gastroenteritis to be more severe (with frequent hematochezia and hospitalization) when it occurs in patients with IBD versus controls, implicating that IBD inflammation exacerbates viral pathology [6].

Murine models show that Norovirus is capable of acting as an inducer in genetically susceptible hosts. In a landmark study, murine Norovirus (MNV) infection of Atg16L1 mutant mice, homozygous for a CD susceptibility allele, induced Paneth cell dysmorphias and inflammatory intestinal disease that resembled human CD. These effects were not seen in uninfected or wild-type mice, indicating a virus-plus-susceptibility gene interaction [12]. Building on this gene-environment interaction, subsequent research demonstrated that ATG16L1 functionally interacts with nucleotide-binding oligomerization domain-containing protein 2 (NOD2) to regulate the autophagic breakdown of invasive bacteria. Notably, loss-of-function mutations in NOD2 or ATG16L1 confer susceptibility to CD [13]. Building on this, recent work has delineated the unique role of NOD2 in Norovirus-induced colitis. Chronic Norovirus infection with strains such as MNoV_S99 and CR6 intensively exacerbated dextran sodium sulfate-induced colitis in wild-type animals, whereas NOD2-deficient animals were protected. Norovirus infection enhanced the expression of NOD2 in myeloid cells, leading to increased phosphorylation of signal transducer and activator of transcription 1 and the release of tumor necrosis factor alpha in response to bacterial peptidoglycan motifs. Virus-induced augmentation of NOD2-dependent microbial recognition created a hyperinflammatory state that exacerbated mucosal injury. Collectively, these findings point toward Norovirus-induced NOD2 activation as a potential environmental "second hit" involved in IBD exacerbations in genetically susceptible individuals [13].

More recently, the interplay between ATG16L1 and Toll-like receptor 7 (TLR7) signaling has also been shown to be critical for antiviral immunity in myeloid cells. ATG16L1 deficiency impaired viral clearance and promoted the expansion of CD8+ tissue-resident memory T cells, thereby worsening colitis severity. Conversely, pharmacologic stimulation of TLR7 signaling decreased mucosal inflammation, suggesting how compromised autophagy-TLR7 signaling can further allow Norovirus to cause intestinal damage in susceptible hosts [14]. In addition, repeated MNV infection has been shown to increase host responses to intestinal injury by amplifying inflammation and altering microbial composition, which results in increased tissue injury. Collectively, these findings validate the hypothesis that norovirus functions as an "environmental second hit," joining host genetic susceptibility factors and microbial alterations as described by the multihit model of IBD pathogenesis [11].

Similarly, MNV infection of Il10-deficient mice, a subclinical colitis model, induced apparent mucosal inflammation and enhanced epithelial apoptosis and barrier disruption, but only in the presence of enteric microbiota, indicating norovirus as a microbial-context-dependent colitogenic pathogen [15]. Experimental studies have revealed that the outcome of MNV-triggered colitis in IL10 -/- (IL10 null) mice is shaped by the microbial context. Mice colonized with altered Schaedler flora (ASF) were found to incur extreme colitis with epithelial injury and barrier abnormalities. In contrast, mice colonized with oligo-mouse-microbiota 12 were found to exhibit low levels of inflammation. Notably, co-colonization with segmented filamentous bacteria protected ASF mice from MNV-induced colitis by promoting barrier defense systems and activating mucosal immunity, illustrating that norovirus-mediated intestinal inflammation is strongly dependent on microbiota [16].

Using these findings to infer human disease, norovirus infection was shown to reorganize the intestinal microbiome of patients with IBD in a manner that favors conditions capable of inducing or sustaining flare-ups. IBD patients who tested positive for norovirus carried a distinctive microbial signature characterized by high abundance of *Ruminococcus gnavus*, Fusobacterium, and Escherichia, organisms generally known to be found in proinflammatory conditions. Although the overall reduction of microbial diversity was not as pronounced as with *C. difficile*, decreased norovirus-positive case diversity correlated with worse clinical outcomes. Norovirus may thus exacerbate IBD by causing barrier loss and immune activation, as well as by selectively enriching proinflammatory taxa, thereby producing a dysbiotic, inflammation-promoting intestinal microbiota [17].

Growing evidence further suggests that the Fucosyltransferase 2 (FUT2) gene is a key risk factor for intestinal disease. Inactivating polymorphisms that generate a nonsecretor phenotype have been shown to enhance susceptibility to IBD; however, in a seeming paradox, they also confer immunity to norovirus (NoV) and RV infection. Immunity results from the absence of histo-blood group antigens (HBGAs), regulated by FUT2, which otherwise act as viral receptors. FUT2 has also been hypothesized to shape gut microbiota composition by affecting HBGA expression, thereby modulating intestinal health [18]. The interaction of IBD with norovirus is more complex than initially believed. Contrary to the postulate that nonsecretors are entirely resistant to norovirus, certain viral types, such as GII.4 and GII.17, are still able to bind to nonsecretor tissues through alternative HBGAs, such as Lewis a (Leᵃ). The binding is firm in regenerative or inflamed mucosa, such as in patients with CD, where glycan structural variations facilitate viral attachment. Such binding may contribute to IBD flares by exacerbating mucosal inflammation or disrupting barrier integrity. Hence, FUT2-dependent HBGA expression not only controls susceptibility to enteric viruses but also intersects with IBD pathogenesis, pointing toward a complex gene-virus-microbiota interaction that warrants further investigation [19].

Although many norovirus-IBD associations occur in predisposed patients, a new case report indicates that norovirus can be an exclusive precipitant in the absence of prior predisposition. Xu and Ning report a child patient with no personal or family history of IBD but who developed biopsy-confirmed UC following a norovirus GII.17 acute infection [20]. The progression from simple viral gastroenteritis to chronic bloody diarrhea and endoscopic signs of colitis in a previously healthy child supports the working hypothesis that norovirus can cause mucosal inflammation de novo [20]. This case provides further support to the view that norovirus is not only coincidental with disease activity but, in single cases, an active precipitant of IBD in immunocompetent hosts.

**Table 1:** Enteric pathogens and IBD (epidemiological studies) UC: ulcerative colitis; CD: Crohn’s disease; hAdV: adenovirus; hAstV: astrovirus; hEV: enterovirus; NoV: norovirus; RVA: rotavirus A; SaV: sapovirus; CMV: cytomegalovirus; ASUC: acute severe ulcerative colitis; EAEC: enteroaggregative *Escherichia coli*; EPEC: enteropathogenic *Escherichia coli*; MDROs: multidrug-resistant organisms; RT-PCR: reverse transcription polymerase chain reaction; IBD: inflammatory bowel disease; GPP: gastrointestinal pathogen panel

Study	Study design	Samples, methods, and materials	Conclusions/discussions
Gebhard et al. [5]	Prospective case control follow-up	266 stool samples, NoV and RVA serology, stool antigens	No significant association. NoV and IBD flare-ups in five out of eight patients with (NoV and RVA) positive serologies correlated with exacerbation symptoms
Khan et al. [6]	Retrospective case control	2,666 stool samples. Norovirus IDEIA assay kit (Dako)	Significant association. NoV-IBD flare-ups nine IBD flare-ups with positive NoV CD (1/23) UC (8/14)
Kolho et al. [7]	Prospective follow-up	50 stool samples RT-PCR: (NoV, hAdV, RVA, hEV, SaV)	No association. No positive enteric viruses during IBD flare-up (0/33), 3/50 (two NoV and one SaV) cases in non-IBD patients
Masclee et al. [8]	Prospective follow-up	286 stool samples RT-PCR (NoV, AdV, RVA, hAstV)	No significant association. In 86 IBD flare-ups, 2.3% hAdV and RVA each; 200 IBD remissions, 0.5% NoV and RVA each, and hAdV (4.5%)
Axelrad et al. [4]	Systematic review	13,231 stool samples. Panel PCR test (22 analytes, including NoV)	Significant association. NoV-IBD flare-ups. Higher prevalence of NoV in both CD and UC (CD; p < 0.05) (UC; p < 0.019)
Axelrad et al. [9]	Cross-sectional database analysis		
Ostrowski and Croft [10]	Retrospective review	147 stool samples. Fecal multiplex PCR testing	No significant association. In 171 patients with ASUC, 33 tested positive for viral pathogens (24 non-CMV) hAdV: 18/146 (10.9%); NoV: 1/146 (0.68%); RVA: 5/146 (3.42%)
Hassan et al. [11]	Cross-sectional observational	95 stool samples. VITEK2 system and FilmArray GPP (22 pathogens, including NoV)	No clear association. NoV-UC flare-ups; NoV in 3.8% active UC cases (low prevalence). Bacterial pathogens (e.g., EAEC/EPEC) dominated (78.7%); mixed infections (70.7%) and MDROs (52.9%)

Stratification by age also further influences these interactions: the immune and microbial status of child patients amplifies the impact of norovirus [21], whereas the majority of adults have some immunity and a stronger microbiome [22]. Primary prevention of norovirus infection is in its budding stages. Currently, there is no FDA-approved norovirus vaccine in clinical use, although a few candidates have shown promising results. Phase 2 data from a bivalent (GI.1/GII.4) virus-like particle vaccine (HIL-214) showed antibody response in both adult and pediatric populations [23].

## Conclusions

Evidence suggests a possible link between norovirus and IBD, either in triggering disease onset or exacerbating flare-ups. However, existing studies, mostly animal-based, are limited by methodological biases and cannot establish causation. Given IBD's complex interplay of immunity, genetics, environmental antigens, and the gut microbiome, simple epidemiological data are insufficient to clarify the role of enteric viruses in its pathophysiology.

Nevertheless, all of this information is valuable as it highlights the gap in the literature and calls for further prospective studies and clinical trials to establish a causal relationship, which can pave the way to a better understanding of the underlying pathology. This can help in developing various treatment guidelines, ultimately leading to better disease outcomes.
